# Clinical Review, and Hospital Reports

**Published:** 1841-10-01

**Authors:** 


### Clinical Review.

### PENNSYLVANIA HOSPITAL.

Statistics of Fractures and Dislocations treated during the Ten Years, from 1830 to 1839, inclusive. By G. W. Norris, M.D.*

Dr. Norris thinks that such a statistical account as this may illustrate beneficially the treatment of fractures and dislocations. Dr. N. first describes the methods adopted in the hospital.

1. *Fracture of the Femur.*

In the treatment of most fractures of the thigh, the straight position is preferred, and the apparatus of Desault modified, is that mostly employed. The improvement consists in the greater length of the outer splint, and the attachment to its lower end of a small block, over a notch in which the extending band passes, in order that the extension be made in a line with the axis of the limb. If the limb can be at once brought down to its natural length, it in all cases should be done on the first application of the apparatus; but when there is so much muscular contraction as to render this very painful, the limb need not be drawn to its full length at first. In these cases it should be extended as much as possible, and, at the second visit of the surgeon, should be seized at the ankle, and slowly pulled downwards, while an assistant tightens and makes fast the extending band. This course is to be repeated until the fragments are perfectly reduced, which may in most cases be readily done at the end of twelve or eighteen hours. No great advantage is believed to be gained by the employment of short splints, or bandages of any sort, applied immediately to the thigh, and their use is dispensed with, as they prevent the surgeon from accurately examining the state of the fracture, and require that the limb should be disturbed in order to re-apply them. A long narrow bag, stuffed pretty firmly with cotton, and covered with buckskin, is used for the counter-extending band; and a double buckskin gaiter, with a thin layer of carded cotton laid over it, or a buckskin band lined with linen, is made use of for the extension. Extension violent enough to cause pain should never be made use of; it ought always to be moderate, steady, and permanent. If constant pain is complained of at any point on which the dressings press, it should be immediately examined and re-adjusted. The restlessness of patients causes any apparatus to be easily displaced, and it is therefore necessary to smooth, tighten, and carefully re-examine it daily. Excoriation of the heel is most frequently produced by want of care in not having the extending band smoothly applied to the part, or by tightening it in too great a degree without having previously drawn down the limb with the hand. Sometimes, however, excoriation is caused by the weight of the foot alone; and in these cases the application of a piece of kid, spread with soap cerate, will mostly prevent it.

We think that sufficient advantage is not usually taken of the buffalo's skin plaster as a means of preventing the bad effects of pressure. Laid on where the bandages are likely to be stringent, it answers admirably in defending the integument. The instep, the heel, and the perineum, should be covered with it. We fancy that the addition of a good boot or foot-piece to the bottom of the

* Amer. Journal of Med. Sciences, April, 1841.

long splint is useful. A common block or cross-bar does not act as uniformly on the foot. Carefully managed, the long splint is an excellent instrument. But we like the short splints with it.

Dr. Norris continues:—In fractures of the femur within the capsular ligament, the application of any splints in the treatment is discarded—the limbs being merely supported by pillows in the extended position. No case has been observed in the period comprised in this report, in which the toes were thrown inwards. Several instances of fractures about the hip-joint, in persons of middle age, have come under notice, in which, even with the most accurate examination and measurement, it was impossible to detect any crepitus or shortening of the limb till after the lapse of one or more days.

2. *Fractures of the Leg.*

In the treatment of fractures of the leg, as in those of the thigh, splints or bandages are rarely applied to the limb. The leg is placed in a fracture-box, upon a well-stuffed pillow, previously covered by a thin oil cloth, in such a manner as to bring the sole of the foot in contact with the foot-board. The fractured bones are then accurately adjusted, and the sides of the box are tied together moderately tight. The foot is securely fastened to the foot-board by means of a strip of bandage, in order to prevent its falling to either side, and the pressure of the pillow is, in the vast majority of cases, quite sufficient to retain the fragments in their natural position. The foot-board of the box is set into its bottom nearly straight, and is made to project beyond the foot, in order to prevent the toes from falling downwards, and thus cause a projection forwards of the upper end of the lower fragment.

We must confess that, in many cases, we prefer the common splints, or an Assalini's box, to the junk. The latter is hot, and, in compound fractures more particularly, inconvenient.

To prevent inflammation, cooling lotions are applied to the limb, and every attention is paid to position. In order to obviate deformity in these fractures when they occur at the lower part of the leg, it is highly important to keep the foot well forwards, and this is best done by placing under the heel some layers of carded cotton.

In very oblique fractures of the leg, where the pressure made by the pillow is not sufficient to prevent the recurrence of deformity after its reduction, permanent extension is kept up by means of Dessault's splints, as in fractures of the thigh.

In fractures of the lower end of the fibula, where the foot is much drawn outwards, the apparatus of Dupuytren, consisting of a conical pad to make pressure over the internal malleolus, and a single splint applied to the inside of the limb, from the upper extremity of the leg to some distance beyond the foot, is employed; but where, as is most generally the case, the tendency to a recurrence of the deformity is not in a great degree, the apparatus commonly made use of for other fractures of the leg, consisting of the fracture-box and pillow, so placed as to exert rather more pressure than usual upon the outer ankle, is resorted to. These fractures are sometimes accompanied by compound luxation of the lower end of the tibia.

In compound fractures followed by suppuration, where the discharge of pus becomes profuse, or where hæmorrhage from the veins or small arteries, either primary or secondary, is troublesome, or is to be looked for, an excellent mode of treatment was introduced into the house many years since by Dr. J. R. Barton. It consists in fixing the limb in a good position in a fracture-box on a bed of dry bran, and surrounding and enveloping it with the same material. This application is soft and pleasant to the patient, makes moderate and very equable pressure, which is increased in proportion to the increase of the hæmorrhage by the bran becoming moistened and expanded, and is unirritating to the wound, at the same time that it may be removed with the aid of a spatula or syringe, and re-applied without causing pain or disturbing in any degree the limb. No mode of dressing that I have ever made use of can be compared to that with bran, in injuries of this kind attended with profuse suppuration, during our extreme hot weather. At this season, the fetor arising from the discharge is often so powerful as to taint a whole ward, and in such cases animalculæ are generated in the course of a few hours, if the wound be in the slightest degree exposed. Clean bran, by covering completely and closely every part of the injured surface, hinders the generation of these animals, and at the same time prevents, in a great measure, the odour that would otherwise arise, by rapidly and effectually absorbing the discharge.

3. *Apparatus for Fractures of the Clavicle.*

The apparatus used at the hospital consists in a pad for the axilla, a ring formed of some soft substance, as a roll of muslin or of buckskin, for the shoulder of the sound side, and a sling for the elbow made of linen, extending half way up the arm, and two-thirds of the way down the fore-arm. To the elbow-piece is attached three strong tapes—one to its upper and posterior part, and one to each anterior extremity. The following is the mode of applying the apparatus; a proper pad being selected and fixed in the axilla, by means of tapes fastened to its upper ends, and passing over to the sound shoulder, the ring or collar is carried up and held on the shoulder of the sound side; the sling is then fitted to the elbow, and after the fracture is reduced by drawing the arm downwards, and pushing the elbow upwards across the chest, the tape on its posterior part is carried over the back and firmly tied to the collar on the opposite side. This done, the surgeon comes round in front of the patient, and makes fast to the collar the tapes attached to the anterior extremity of the elbow-piece. These are to be drawn tight enough to throw the shoulder sufficiently outwards and upwards to remove all deformity. The hand is then supported in a sling, or by a strip of bandage fastened to the collar. The whole apparatus is to be re-examined and tightened daily. The chief indications in the treatment of fracture of the clavicle are perfectly fulfilled by the use of this apparatus; the pad in the axilla throws the shoulder outwards, at the same time that the drawing up of the elbow by the linen bag throws it upwards and backwards. Besides this it is simple, requires no bandaging, and leaves the part injured at all times open to inspection. The apparatus, too, can readily be applied in females, in whom it is all important to obviate deformity. The apparatus was contrived and introduced into the practice of the hospital in 1828, by Dr. Fox, then house surgeon, since which time it has been constantly employed.

4. *Fracture of the Humerus.*

In fractures situated at the middle of the bone, three or four pasteboard splints are commonly made use of—the outer extending from the top of the shoulder to the external condyle, the inner reaching from the axilla to just above the internal condyle, and the anterior one sufficiently long to reach to the bend of the arm. The roller is applied, as in all other cases, from the fingers up, and the fracture being reduced, and the splints fixed as mentioned, is returned and fastened over them. The arm may then be bound to the body by a broad bandage, or can be left free, supported by a sling. In patients who are very restless, the roller soon becomes loose about the fore-arm and elbow, and necessitates a frequent re-application of it. To prevent, in a measure, this almost daily renewal, the use of an angular board splint well padded, and extending from the axilla to the hand on the inside of the arm, is sometimes substituted; one or more short splints being at the same time applied above the elbow. The fragments by this means are held perfectly in place, and the angular splint, by holding the fore-arm at rest, keeps the bandage well and evenly applied for a much longer period than is otherwise possible. During the cure, the angle of the splint should be occasionally changed, in order to prevent any degree of stiffness at the elbow. This mode of dressing is applicable to fractures below the insertion of the deltoid only; for fractures situated high up in the bone it would be manifestly improper.

5. *Fractures of the Elbow.*

One of the most common of these, after fractures of the inner condyle, is that in which two fractures and three fragments are present, the humerus being broken transversely just above the condyles, and these last separated longitudinally. All fractures about this part are very troublesome and serious accidents, and to treat them well, requires extraordinary care and attention, whatever method of treatment may be made choice of. Two rectangular splints applied to the inner and outer sides of the arm, and extending from its upper part to the ends of the fingers, are frequently employed with us. When these are used, the angles of the splints should be frequently changed to prevent deformity and stiffness of the joint—those first applied being removed after ten or twelve days, and replaced by others of an obtuse angle. Another method of treatment, which is sometimes pursued at our hospital in fractures about this joint with very satisfactory results, consists in the application of a single board splint applied to the front of the arm. This should be of the width and shape of the limb, well padded, and extending from the upper part of the arm to the ends of the fingers. At first a right angled splint may be used, but at every dressing, (and after the first few days they should be frequent,) it is to be changed for a more obtuse angled one, until finally the arm can be brought perfectly straight. The obtuse angled splints are then recommenced with, and gradually replaced by others less obtuse, until the limb is again brought to a right angle. This plan carefully pursued, will generally prevent deformity, at the same time that it is of more easy application, and more effectually hinders the occurrence of anchylosis than the common mode of dressing.

6. *Fractures of the Fore-arm.*

These accidents are treated by means of a roller and two splints, applied in the usual manner. Fractures of the lower end of the radius, which, it may be remarked, are very frequently mistaken for simple sprains, are treated also with two splints; the inside one extending from the upper part of the arm beyond the ends of the fingers, while that on the outside passes below the knuckles. In these, as in all other cases in which a simple fracture communicates with, or is in the immediate neighbourhood of the wrist or elbow joints, the dressings should be removed at the end of ten or twelve days, and after the joint is gently exercised, are to be re-applied. This should be repeated, at farthest, every second day. The same rule should be observed in all cases in which the fore-arm is confined in two long splints, as otherwise great rigidity of the wrist-joint occurs, which is annoying to the patient, and requires a very long time for its disappearance.

7. *Fractures of the Cranium.*

In cases of simple fractures of the cranium with depression of bone, but unaccompanied with symptoms of compressed brain, the trephine has not been resorted to, while, in instances of compound fracture, attended with depressed fragments, even where no symptoms of compression existed, it has been the general practice to remove the portions driven in.

8. *Number of Fractures and their Relative Frequency for twelve years.*

| | Number. | Cured. | Relieved or by request. | Died. | Amputations. |
|---|---|---|---|---|---|
| Fractured thigh | 118 | 92 | 9 | 17 | 2 |
| Fractured leg | 293 | 241 | 11 | 41 | 14 |
| Fractured cranium | 46 | 14 | 2 | 30 | |
| Fractured patella | 16 | 14 | 2 | | |
| Fractured spine | 8 | | 1 | 7 | |
| Fractured sternum | 5 | 4 | | 1 | |
| Fractured clavicle | 84 | 75 | 7 | 2 | |
| Fractured arm | 250 | 216 | 22 | 12 | 9 |
| Fractured fingers | 9 | 8 | 1 | | |
| Fractured scapula | 10 | 8 | 1 | 1 | |
| Fractured elbow | 2 | 1 | | 1 | |
| Fractured nose | 3 | 3 | | | |
| Fractured jaw | 19 | 12 | 1 | 6 | |
| Fractured pelvis | 5 | 5 | | | |
| Fractured foot | 12 | 10 | 2 | | 4 |
| Fractured ribs | 46 | 39 | 2 | 5 | |
| Fracture | 2 | 2 | | | |
| Compound fractured pelvis | 1 | | | 1 | |
| Compound fractured knee | 2 | 1 | | 1 | 2 |
| Compound fractured thigh | 15 | 4 | 1 | 10 | |
| | 946 | 749 | 62 | 135 | 31 |
| Un-united fractures | 13 | 9 | 2 | 2 | |
| Total, | 959 | 758 | 64 | 137 | 31 |

No instance of artificial joint followed the treatment for fracture during the ten years included in the report. All the cases observed there, during that period, having been sent to the institution from distant parts.

9. *Dislocations treated during* 10 *years.*

| | Number. | Cured. | Relieved or by request. | Died. |
|---|---|---|---|---|
| Dislocated shoulder | 49 | 45 | 3 | 1 |
| Dislocated hip | 17 | 13 | 3 | 1 |
| Dislocated astragalus | 1 | | | 1 |
| Dislocated cervical vertebræ | 1 | | | 1 |
| Dislocated jaw | 2 | 2 | | |
| Dislocated ankle | 2 | 1 | | 1 |
| Dislocated elbow | 7 | 7 | | |
| Dislocated wrist | 4 | 4 | | |
| Dislocated clavicle | 3 | 3 | | |
| Dislocated radius | 1 | 1 | | |

| | Number. | Cured. | Relieved or by request. | Died. |
|---|---|---|---|---|
| Dislocated finger | 1 | 1 | | |
| Dislocated great toe | 1 | 1 | | |
| Compound dislocated thumb | 2 | 1 | 1 | |
| Dislocation and laceration | 1 | | | 1 |
| Dislocation and fracture | 1 | 1 | | |
| Dislocation | 1 | | 1 | |
| Total, | 94 | 80 | 8 | 6 |

With the exception of a few of the luxations of the shoulder and hip, the cases included in the following table were of recent occurrence, and in all of these cases the bones were reduced without accident of any sort. Two out of the three luxated shoulders marked as discharged by request were incurable.

One of these was a case of dislocation of the humerus forwards of sixty-two days' standing. The pullies were used ineffectually. The other was a forward luxation of three months' standing. No force could reduce it.

Among the cases of dislocation at the shoulder cured, are those which had been out twenty-six, forty-five, fifty-three, thirty-one, ten, twenty-one, and thirty-one days, respectively.

The only instance of luxated hip of long-standing, in which attempts at reduction were deemed proper, was that of a lad, ætat. 12. The signs of dislocation, upwards and backwards on the ilium, of the right femur, were well marked, with shortening of the limb to the extent of two inches. The accident had happened fourteen weeks previously, and its nature had been mistaken by the person called to visit him. The injury had been produced by a fall from a height, and the head of the bone was to a certain extent moveable by rotating the limb. After the application of the pullies he was bled largely from the arm, and took a solution of tartar emetic, by which means his system became completely relaxed. Pretty strong extension was then made from above the knee, and after this had been kept up near half an hour, the head of the bone was drawn outwards by means of a towel, and the knee rotated. During these efforts a sudden crack was distinctly heard. The apparatus was immediately removed. The boy was now exceedingly prostrate. Much more motion than before the trials existed at the hip; the toes could be turned much further outwards, and the knees could be to a certain extent separated. The boy did not complain of pain on rotation, and took a few steps upon the limb some time after being released. It was at first feared that a fracture at the neck of the bone had been produced, but repeated close subsequent examinations proved this not to be the case. He left the institution twenty-two days after the trial, with more motion in the limb than he had previously had.

There was a case of dislocation of the cervical vertebræ, unattended with fracture. The yellow ligaments and the ligamentous fibres, holding together the oblique processes, were ruptured, and the fifth cervical vertebra was thrown forwards upon the sixth. Examined in front, the vertebral ligaments were found also to be ruptured, and the inter-vertebral substance torn up, so that the body of the fifth was completely separated from, and projected over the sixth. Accurate examination, after the removal of the upper part of the spinal column, proved that no fracture existed, and that the injury consisted in a simple displacement of both body and processes of the vertebræ.

---

### SHATTUCK ON THE VITAL STATISTICS OF BOSTON.*

Our American contemporary contains an interesting paper on this subject. We shall allude to some of the principal points.

The system of registration of births, deaths, and marriages, would seem to be very defective in America. But an amendment is purposed.

The population of Boston was estimated at the beginning of the last century to have been about 6,750, and the annual deaths to be 230—one in 29.3, or 3.4 per cent. The deaths from 1705 to 1714, inclusive, were 3,341, and from 1715 to 1724, 4,350, giving an annual average of about 1 in 24, or 4.09 per cent.

The lowest mortality was in 1755 to 1764, being 514—one in 34, or 3.24 per cent. of the population, annually; the highest was in 1745 to 1754, being 671—one in 23, or 4.26 per cent. This is just double the mortality, which prevailed in 1826 to 1835, being then only 2.13 per cent. The lowest mortality in any

---

* Amer. Journ. of Med. Sciences, April 1841.

single year was 407, or 1 in 38, in 1763; the highest 909, or 1 in 14, in 1730, and 1009, or 1 in 15, in 1752.

The mortality of the black, was much greater than that of the white population. In the first period mentioned in the table, it was as low as at any time. One in 18, or 5.64 per cent. of the black population, died, showing a difference of 2.56 per cent. as compared with the mortality of the whites. The highest mortality among the blacks was in 1725 to 1734, being 1 in 12, or 8.64 per cent. These are very striking facts, but are accounted for, in some measure, by the prevalence of the small-pox and other epidemics, which often visited the town at that time, and which seem to have been peculiarly fatal to the black population. In fact, the liability to death by this disease among the blacks, was about 50 per cent. greater than among the whites, when taken in the natural way; and more than three times as great, when taken by inoculation.

It appears from a table that the population of Boston was, in 1790—18,038, and in 1837, 80,325. The greatest increase of the population was from 1820 to 1825, being 14,983, equal to an annual increase of 6.92 per cent., or 1 in 14. The least increase was in 1825 to 1830, being only 1.06 per cent. annually. The whole increase from 1790 to 1837, was 445.3 per cent., or doubling the first mentioned number about 4½ times. There were 201 square yards to each inhabitant in 1790, and 49 in 1837, being an increased density of 5 to 1.

The proportion of white to coloured population has been about the same at each of the enumerations, excepting the last two, when the whites had increased, as will appear from the following statement:

| Proportion. | In 1790. | In 1800. | In 1810. | In 1820. | In 1825. | In 1830. |
|---|---|---|---|---|---|---|
| Of whites, | 95.78 | 95.30 | 95.60 | 95.98 | 96.71 | 96.95 |
| Of coloured, | 4.22 | 4.70 | 4.40 | 4.02 | 3.29 | 3.05 |
| | 100.00 | 100.00 | 100.00 | 100.00 | 100.00 | 100.00 |

This shows that in 1790, of the whole population, 95.78 per cent. were white, and the remainder 4.22, were coloured. In 1830, the proportion of whites had increased, and the coloured decreased, 1.17 per cent.

From a mortality table, it appears that the least in one year was in 1827, being 939, one in 63, or 1.57 per cent., and the greatest in 1821, being 1,321, one in 35, or 2.85 per cent. The average annual deaths were 813, from 1811 to 1830—one in 47, or 2.09 per cent., 1147 from 1821 to 1830—one in 49, or 2.05 per cent., and 1,552 from 1831 to 1839—one in 46, or 2.14 per cent. showing a small increase in the force of mortality.

The value of life would seem to have materially increased in Boston. Nearly 100 years ago there was 1 death to about 30 or 35, or 3 per cent. of the population. Now according to table IV. there is 1 in about 45, or 2.10 per cent. of the population. This shows a very great improvement in the relative value of life.

Mr. Shattuck gives a table showing the population of Boston for 1830, the deaths for 10 years, 1826 to 1835, and the proportion of the annual deaths to 100 constantly living in Boston and Carlisle. From this table it appears that the proportion of deaths to the living is greater in Carlisle that in Boston under the age of 20, and between the ages of 80 and 90. At the other ages it is greater in Boston. Between the ages of 50 and 60, and 90 and 100, there is the greatest difference, being in the former 1.23, and in the latter 2.32. The mean of all ages is 2.13 per cent. in Boston and 2.50 per cent. in Carlisle, showing a difference of .37 in favour of the former. This presents the law of mortality in Boston, as accurately as it could be done from any data at present existing. It is, however, difficult, if not impossible, to determine it with perfect precision by any general statement concerning a population so changeable as that of Boston.

Mr. Shattuck compares the relative mortality of the different periods, 1811 to 1820, 1821 to 1830, and 1831 to 1839. This comparison presents some very striking facts, and shows that, although the average value of life is greater now than during the last century, it is not so great as it was twenty years ago; that it was at its maximum in 1811 to 1820, and that it has since somewhat decreased. It appears that 33.64 per cent. of the deaths in 1811 to 1820 were under 5 years of age, 37.04 per cent. in 1821 to 1830, and that 43.09 per cent. in 1831 to 1839, showing a gradual increase of the relative mortality under that age, and between the first and the last given period, a difference of 9.45 per cent. or a proportional increase of mortality of 28 per cent.!

It is a melancholy fact, and one which should arrest the attention of all, that 43 per cent. or nearly half of all the deaths which have taken place in Boston during the last nine years, are of persons under 5 years of age; and the proportional mortality of this age has been increasing.

The causes of this increasing and alarming mortality should be investigated, and, if possible, removed. More luxury and effeminacy in both sexes prevail now than formerly; and this may have had some influence in producing constitutional debility, and the consequent feeble health of children. The nursing and feeding of children with improper food is another cause. The influence of bad air in confined, badly located, and filthy houses, is another and perhaps the greatest. Epidemic diseases which are particularly prevalent among children have increased. It will hereafter be shown that scarlet fever has prevailed very much the last nine years, and has increased the mortality. In the period 1811 to 1820, this disease produced 13 per 1000 of the whole deaths. In 1831 to 1838, it produced 489. Other infantile diseases have also increased. These considerations would, perhaps, sufficiently account for the increased mortality under 5 years of age.

*Mortality of Different Sexes.*—From tables, it appears that there were 180 more female than male deaths in 1811 to 1820; 424 in 1821 to 1830, and 341 in 1831 to 1839. There were four years in the first period, and three in the last only, when there were more male than female deaths. The proportion of the different sexes will appear from the following statement.

| | | Deaths. | Average. | Proportion. | | |
|---|---|---|---|---|---|---|
| 1811 to 1820 | Males | 4156 | 415 | 51.11 | as 100 | or 104.59 |
| | Females | 3976 | 397 | 48.89 | to 95.66 | 100. |
| | Total, | 8132 | 813 | 100.00 | | |
| 1821 to 1830 | Males | 5947 | 594 | 51.85 | as 100 | or 107.67 |
| | Females | 5523 | 553 | 48.15 | to 92.87 | 100 |
| | Total, | 11470 | 1147 | 100.00 | | |
| 1831 to 1839 | Males | 7457 | 828 | 51.17 | as 100 | or 104.79 |
| | Females | 7116 | 790 | 48.83 | to 95.42 | 100 |
| | Total, | 14573 | 1618 | 100.00 | | |

The proportion of male and female deaths to the population in the years when the census was taken, was as follows:—

| | One in | | Excess. | Proportion per cent. | | Excess. |
|---|---|---|---|---|---|---|
| 1820 | Males 41 | Females 44 | 3 | Males 2.41 | Females 2.27 | 14 |
| 1825 | „ 41 | „ 43 | 2 | „ 2.32 | „ 2.27 | 5 |
| 1830 | „ 54 | „ 65 | 11 | „ 1.83 | „ 1.52 | 31 |
| 1835 | „ 39 | „ 48 | 9 | „ 2.54 | „ 2.07 | 47 |

This statement shows that the agents of death are uniformly more active with male that female life.

From another table, it appears that at the ages under 10 years, and between 30 and 60, more males than females die, the proportion rising in 40 to 50, as 56.99 to 43.01 per cent. At the other periods specified there are less male than female deaths; the difference after the age of 60 continually increasing, until 90 to 100, when it was as 27.18 to 72.82 per cent. But it is certain that there were a greater number of males than females at any age among the living population. A comparison of these with the deaths will show that at certain ages a greater proportional mortality prevails among males, and at other ages among females.

*Mortality of the different Seasons of the Year.*—It appears that the months of August, September, and October, have the highest mortality. December is number 4 in each period; November 5 in the last, and 6 in the others. June has uniformly the least mortality. If the proportions are arranged according to the seasons of the year, they will be as follows:—

| | 1811-1820. | 1821-1830. | 1831-1839. |
|---|---|---|---|
| Winter.... | 2.801 | 2.775 | 3.000 |
| Spring.... | 2.842 | 2.825 | 2.622 |
| Summer .. | 2.807 | 2.996 | 2.800 |
| Autumn .. | 3.550 | 4.434 | 4.578 |
| | 12,000 | 12,000 | 12,000 |

Mr. Shattuck has endeavoured to arrange the facts for one period, 1821-1830, to ascertain what influence the seasons have upon the mortality of different ages. He has given the whole deaths during the time, the mean or average of each month at each age, and the difference from this mean, placing the sign minus, when the mortality of the month at any age was less, and plus when it was greater, than this mean. The table itself is too long for us, but the following abstract willl be intelligible.

| | Under 20 years. | 20 to 60. | 60 and upwards. |
|---|---|---|---|
| Spring...... | — 205.50 | + 43.75 | — 3. |
| Summer .... | + 56.50 | + 62.75 | — 49 |
| Autumn .... | + 366.50 | + 70.75 | + 10 |
| Winter...... | — 222.50 | — 35.75 | + 52 |

From this statement it appears that the seasons have the greatest influence on the mortality of persons under the age of 20 and over that of 60—the summer and autumn being most fatal with the former, and winter with the latter. This is the only general law we can deduce from the tables.

*The Still-born.*—The proportion of the still-born to the whole burials was 1.82 per cent. in the second, and .65 per cent. in the third period, more than in the first. By table VIII. it appears that the month producing the highest proportion was August, and that March was the next highest. There appears, however, to be less variation, in regard to the seasons, in these than in the other deaths.

*Diseases.*—The mortality tables of Boston would appear to be defective to a very great degree, in regard to the particular cause of death. Mr. Shattuck makes the following sensible remarks:—

"It has been considered sufficient by many writers on this subject to prepare the tables, so as to exhibit the number of the deaths by each disease for certain definite periods of time given. But this information appears to fall short of the result which ought to be presented in such tables. To render them useful, a comparison should be made between the number of deaths by each disease, and the whole number of deaths in a certain given period, and this result should be again compared with a similar result concerning other periods. In this way the prevalence of any particular disease compared with that of other diseases at the same period, and with same diseases at different periods, may be at once seen, and a judgment formed from the per centage what proportion of deaths that particular disease occasions, and whether it be on the increase or decrease. The sex, age, and place of nativity of the diseased, and the season of the year in which the deaths occurred, are not stated in connection with the diseases in our printed tables, but they should be. The fatality of disease depends much on the age of the patients, and it is not the same in childhood, manhood, and old age, nor with the different sexes, and in the different months of the year. It is very important to know all such facts in relation to each disease, and the danger that man has to encounter in all ages, and under all circumstances. It would also be important, if practicable, to know the number of deaths by each disease in proportion to the population, distinguishing them according to their ages. When facts like these are known they will lead to inquiries into the causes, which have produced an increase or diminution of disease, under different circumstances, and lead to the adoption of the proper remedies."

Mr. Shattuck divides the years from 1811 to 1839 inclusive, into three periods, the first from 1811 to 1820, the second from 1820 to 1830, and the third from 1830 to 1839 inclusive.

*Fevers.*—The deaths by fevers of all kinds were 749, 604, and 721, or 88.4, 52.7, and 49.5 per 1000 of the whole deaths in the respective periods, showing a decrease of 35.7 in the second, and 3.2 in the third. By looking at the different fevers in the tables it will be perceived, that typhus has produced the greatest number of deaths, but still it has very much decreased; being 623, 458, and 611, or 73.5, 39.9 and 41.9 per 1000, showing the last eight years a small increase on the previous 10, but not more than half the proportion of the period 1811 to 1820. The greatest number in one year was 119, in 1818. Ten cases of yellow fever occurred in 1816. There is evidently an error in the numerical statement relating to the third period.

*Eruptive Fevers.*—The diseases of this class occur very irregularly. They have, however, increased. There occurred 64, 402, and 1402, or 7.5, 35.1, and 96.2 per 1000 in the respective periods, the last period showing more than 13 times the mortality of the first. Each of the diseases, excepting *thrush*, shows an increased mortality. *Erysipelas* has increased from 1, in the first, to 65 in the last period. *Measles* was very fatal in 1821, 1825, 1829, 1832, and particularly in 1835, when 188 died of this disease: 28, 332, and 340, or 3.3, 28.9, and 23.1 per 1000 of the whole deaths occurred from this disease in the respective periods. Cases of scarlatina have increased since 1821 to 1830, from 13 to 489! It has become one of the most fatal of the eruptive fevers. The suddenness of its attack, the irregular mode of its operation, and its generally fatal termination, has rendered it one of the diseases most to be dreaded. The greatest number in one year was in 1839, when 222 died. The next greatest was 200 in 1832. To the prevalence of this disease may be attributed, in some measure, the increased mortality of children under five years of age. The recorded cases of *Small-pox* have been principally at the quarantine establishment at Rainsford Island. It never prevailed in the city, as an epidemic, during the period under review, until the autumn of 1839. It then spread generally through the city, and produced 60 deaths before the close of the year.

A table is given of the mortality in the different periods from cholera, croup, dysentery, and hooping-cough. That of all has been upon the increase.

| Diseases. | 1811—1820. Number. | Ratio per 1000. | 1821—1830. Number. | Ratio per 1000. | 1831—1839. Number. | Ratio per 1000. |
|---|---|---|---|---|---|---|
| Cholera, | 122 | 14.4 | 149 | 12.9 | 407 | 27.9 |
| Croup, | 43 | 5. | 245 | 21.3 | 376 | 25.9 |
| Dysentery, | 115 | 13.5 | 429 | 37.4 | 372 | 25.5 |
| Hooping Cough, | 78 | 9.2 | 184 | 16.0 | 256 | 17.5 |

The total of this class of diseases was 380, 1031, and 1499, or 44.9, 89.9, and 102.9 per 1000 in the respective periods.

*Diseases of the Nervous System.*—It appears that the whole of the diseases of this class have been 562,980, and 1515, or 66.4, 85.4, and 104. per 1000 in the different periods, showing a slight increase. The entries under each class, excepting epilepsy, insanity, and tetanus, also show an increase. *Insanity* has not appeared to increase, though some allowance should be made for the patients afflicted with this disease, who go to the Lunatic Asylums at Worcester and Charlestown, and sometimes die there. If these were considered in our reports they would probably show a different result, and a slight increase of the disease. The following statement shows the proportional prevalence of the three principal diseases of this class.

| Diseases. | 1811—1820. Number | Ratio per 1000 | 1821—1830. Number | Ratio per 1000 | 1831—1839. Number | Ratio per 1000 |
|---|---|---|---|---|---|---|
| Apoplexy, | 109 | 12.8 | 107 | 9.3 | 162 | 11.1 |
| Convulsions, | 229 | 27. | 309 | 26.9 | 419 | 28.7 |
| Hydrocephalus, | 86 | 10.1 | 270 | 23.6 | 498 | 34.1 |

*Diseases of the Organs of Respiration.*—In the different periods under consideration, 2460, 2802, and 3214 deaths, or 290.5, 244.8, and 220.5 per 1000, were caused by this class of diseases. This indicates a decrease of 70 per 1000 from the first to the last period. The following table will show the comparative prevalence of the principal diseases:

| | 1811—1820. Number cases | Ratio per 1000 | 1821—1830. Number cases | Ratio per 1000 | 1831—1839. Number cases | Ratio per 1000 |
|---|---|---|---|---|---|---|
| Pleurisy, | 35 | 4.1 | 40 | 3.4 | 83 | 5.7 |
| Phthisis, | 1891 | 223.3 | 2054 | 179. | 2066 | 141.7 |
| Pneumonia, | 436 | 51.4 | 580 | 50.5 | 937 | 64.2 |

The leading disease of this class, and indeed of all classes, is phthisis, or consumption. From these tables it appears to have decreased over one-third from the first to the last period. Entire reliance, however, should not be placed on this statement. There is so much indefiniteness in the application of the term *consumption*, as well as many other terms in our bills, that it should be regarded only as an approximation to the truth. The more accurate diagnosis recently observed has probably given a different classification to many cases, from that assigned to them in the first period. Consumption is, however, a most formidable disease, not in Boston peculiarly, but in all cities and country towns. Sufficient facts are known to show, that from *one-fourth* to *one-seventh* of all the deaths in the Northern and Middle states, and perhaps throughout the whole Union, and the civilized world, are caused by consumption. Mr. Shattuck justly observes, that the only chance of materially diminishing the fatality of this malady is by making the people acquainted with the causes of it, and instructing them in measures of prevention.

*Diseases of the Organs of Circulation.*—These diseases have increased, being 22, 81, and 191, or 2.5, 7. and 13.1 per 1000. Of the whole of this class

24, 90, and 200, or 2.9, 7.9, and 13.7 per 1000 occurred in the different periods.

*Diseases of the Digestive Organs.*—The following statement will show the comparative prevalence of some of the principal diseases of this class:

| | 1811—1820. | | 1821—1830. | | 1831—1239. | |
|---|---|---|---|---|---|---|
| | Number cases | Ratio per 1000 | Number cases | Ratio per 1000 | Number cases | Ratio per 1000 |
| Enteritis, | 6 | .7 | 162 | 14.1 | 320 | 21.9 |
| Teething, | 39 | 4.6 | 83 | 7.2 | 247 | 16.9 |
| Worms, | 21 | 2.5 | 26 | 2.2 | 51 | 3.5 |

Most of the other diseases specified have decreased, excepting those of the liver, and the other organs mentioned under the general head, and included under *Disease.* These have greatly increased. The whole number of cases were 231, 644, and 1107, or 27.3, 56.1, and 76, in the different periods.

*Of the Urinary Organs—Kidneys, Ureters, Bladder, Urethra.*—Under *Stone* are included all who died of stone or gravel. In the first period there died of this disease 1 in 1411 of all diseases, in the second 1 in 546, and in the third 1 in 2082. Of all the diseases of this class 9, 30, and 22, or 1.1, 2.6, and 1.5 per 1000 occurred in the respective periods.

*Of the Organs of Generation.*—Under *Childbed* are included cases of "puerperal fever," 63, 121, and 175, or 7.4, 10.5, and 12. per 1000, in the respective periods, occurred by this disease; and 64, 132, and 192, or 7.6, 11.5, and 13.2 per 1000 of the whole deaths of this class.

We pass over diseases of more vague determination, trusting that a better system of registration will progressively diminish these in all countries. Mr. Shattuck adds:—

"From this view of the causes of death in Boston it appears that 1193, 2037, and 3622 cases, or 140.8, 177.7, and 248.6 per 1000 of all the deaths were from epidemic, endemic, and contagious diseases; and that 7275, 9433, and 10951 cases, or 859.2, 822.3, and 751.4 per 1000 of all the deaths were from sporadic diseases. This shows an increase of the first, and a decrease of the second division of diseases, in the respective periods. If, as has been stated, the great criterion of health is the comparative prevalence of one or the other of these two great divisions of diseases, it follows that Boston is not now quite as healthy as it was twenty or thirty years ago. This fact, I think, may be inferred also from other investigations given in this article."

We are glad to perceive that our transatlantic brethren are taking up the subject of vital statistics. Nothing is more calculated to advance our knowledge of the great causes of disease, of the circumstances that affect the public health, of the means to be taken to improve it, and to force these important questions on the attention of the legislature and the executive.

---

### GUY'S HOSPITAL.

### CLINICAL OBSERVATIONS BY MR. MORGAN.*

#### 1. *The Nockemorfs of Guy's.*

Be careful what you say respecting the nature and treatment of a patient's

---

* Prov. Med. and Surg. Journ. April 10, 1841.

case, either in his presence, in the presence of his relations, friends, or attendants; or under any circumstances which may make him or them acquainted with what it is quite unnecessary for them to know. Ever be careful of eavesdroppers. Now do not mistake me. The truth—nothing but the truth—ought always to be spoken, but there is a time and a place for all things, and it is not always necessary to speak the *whole* truth to patients. Deception is always despicable; but, for the welfare of our patients, concealment is sometimes necessary. The concealment to which I allude is neither criminal nor improper—it consists in avoiding the abuse of that unruly member, the tongue. Now I will illustrate and explain what I mean to insist upon. In the room set apart, in this noble institution, for those who are anxious to become our patients here, on our taking-in days, you will all of you have free access to them before they are seen by the medical officers. Now you may interchange thoughts and opinions in the *hearing* of those applicants for relief, which are not only improper, but injurious to the interests not only of them but of yourselves. Often and often has a patient been driven from the "taking-in" room of a hospital, from having heard the tittle-tattle of medical students in reference to his case. If the case be an urgent one, is not this injurious to the welfare of the listener? But whether urgent or not, we are liable to the loss of the opportunity of showing you the treatment of disease, either by local or constitutional means, by the very great imprudence I am alluding to. Is not this injurious to your interests as well as theirs? Let me illustrate what I mean by one or two examples. A patient, in one of our wards, labouring under serious disease, was asked by one of the pupils where he came from. He replied, from Wisbeach, "O," said the pupil, "my good fellow, you will never see Wisbeach again;" and this had such an effect upon him that he immediately left the hospital. Let us take another instance from the taking-in room. A delicate, hysterical female presents herself, with a small tumor in the breast, which she wishes to have removed by the most lenient measures—as she herself would say *dispersed.* Now, this is what is commonly considered as an interesting case, and a group of pupils collects around the patient. The dresser of Mr. A. examines the breast, and says, "O! here is a nice case for operation. Two semi-elliptical incisions, embracing the whole surface, and then cleanly dissected out." "No," says a pupil of Mr. B., "it would be done much better by a crucial incision, and dissecting back the flaps. B. always takes away the whole of the breast, but generally leaves the nipple." Another young gentleman observes, "That's a case for C., he stands at nothing; he'd have it out in a minute, root and branch;" and the poor girl, now thoroughly frightened, hurries away from the tender mercies with which she is threatened, as horrible in her imagination as the tortures of the Spanish Inquisition.

2. *Never tell a Man you mean to give him Pain.*

Surgeons are sometimes equally in error with their pupils, and communicate to their patients what mere patients have no business to know, until they have found it out themselves. There can be no necessity for telling a subject for operation, that you are going to give him pain; common sense will teach him this. Let him know when you are going to commence an operation, and encourage him to bear it firmly; but do not remind him of what he has to undergo. It can do no good, and may possibly unnerve a strength of mind and body previously wound up to the sticking point. I remember an Irishman, who had a fracture of his frontal brone with depression, but no very urgent symptoms had then supervened. He was brought into the operating theatre, for the purpose of being trephined to raise the depressed portion of bone, and Mr. Cline, who was constantly in the habit of preparing his patients for the knife in the manner I have been deprecating, said, "Now, my good fellow, you must be firm, very firm, for I am about to give you a very great deal of pain." When the Irishman leaped from the table, exclaiming, "Och! by Jasus, but you won't though;" rushed from the theatre, and was never seen in the hospital again.

We certainly think that Mr. Cline was rather *green* in telling the man that he should give him a good deal of pain. The common address is—"Now, my good fellow, I'm going to give you a *little* pain." But if a man is inclined to run away, he is as likely to bolt when he is told you are going to begin, in one way as in another.

3. *Mind what you are about in Consultations.*

A very moral young gentleman had been undergoing some violent exertion by which he had strained the interior of the walls of the abdomen. He complained of a good deal of stiffness and pain above the pubes, and the apothecary whom he consulted ordered leeches, followed by fomentations and poultices. There were only the mother and an old nurse in the house with the youth, and they became naturally suspicious at leeches and poultices to a part which they were not allowed to examine. Mr. Hunter was called in, and the nurse, who was very curious to determine exactly how matters stood, applied her ear to the door, and overheard him telling the apothecary that there was a severe strain of the abdominal muscles. She directly ran to her mistress, "O marm, its just as I thought!" "Why, what's the matter?" "O marm, Master Thomas has been and strained his abominable muscle!" (loud laughter.) I may give you an instance also to show how cautious you should be in your manner of speaking before your patient as to the nature of his disease. A patient of Dr. Babington, who had some very obscure abdominal swelling, saw that considerable doubt was entertained as to the precise disease under which he laboured, and consulted one of my colleagues. He saw Dr. Babington some time afterwards, and said, "O, sir, I wish I had consulted Mr. —— sooner. He saw directly what was the matter." "Why," replied the Doctor, "what did Mr. —— say it was?" "O, sir, why he said directly it was a *tumour*." So you see, gentlemen, how much may be gained by a few generalities in your professional consultations; but I repeat, be careful in speaking before your patients—few have had occasion to repent having said too little—many of having said too much.

---

### CASES FROM THE EARLY NOTE BOOKS OF SIR ASTLEY COOPER.*

1. *Salt-water Poultice.*

This poultice is an excellent application where absorption of extravasated matter is required, or when it is wished to occasion an abundant discharge, to quicken the action of a deep-seated abscess. Miss —— bruised the middle and fore-part of the leg, occasioning a large ecchymosis and a small sore. The inflammation of this sore spread to the ecchymosed part, which suppurated, though slowly and imperfectly. Tired of their first surgeon, as she had been confined for three months, I was sent for. A small sore at this time appeared on the shin, and a swelling on the outside of the leg, on pressing which, matter was discharged at the sore.

I ordered her a salt-water poultice, a table-spoonful to a pint. In three days the swelling was lessened, but the discharge increased. I then ordered two table-spoonfuls of salt to a pint of water, and in two days the swelling was gone, and the discharge had nearly ceased.

The first proportion was then ordered, and it rapidly filled up and healed.

We cannot say that we are so fond of salt-water poultices. We have seen

---

* Provincial Med. and Surg. Journal, August, 1841.

them give rise to much irritation, and convert a trifling ulcer into a troublesome one. This has happened several times within our observation in cases of ulcers connected with scrofulous glands.

### 2. *Want of Synovia.*

A servant of Mr. ——, Queen Street, had a stiffness come on in both her knees, without any visible external appearances of disease. The bones grated upon moving them over each other in the joint, and if she used her legs she complained that considerable heat was occasioned by it. No application was of any service to her.

### 3. *Imperforate Jejunum.*

A child was born apparently healthy, but was found to reject, in a few minutes, everything that it took into its stomach. A bougie was passed into its rectum, and I found no obstruction. It was ordered castor oil and various other purgatives, which almost immediately were returned. It wasted away rapidly, and died twelve days after its birth.

*Dissection.*—I opened it on the day of its death. On cutting into the abdomen, an intestine of great size presented itself—two inches in diameter—which was found to be imperforate. It was the jejunum, which had this blind extremity; it was placed about ten inches from the origin of that portion of the intestines.

The portion of gut, naturally next to this, but still in this infant entirely separate from it, was not larger than small whipcord; and this, after passing six inches, was obstructed in the same manner. The lower part of the ilium and the large intestines, which were without disease, were, however, not larger than a goose-quill, from having never been distended. The gall-bladder was quite empty, but the imperforate intestine was full of bile and medicine.

---

### Clinical Remarks of M. Andral on Fever and Inflammation.*

### 1. *State of the Blood in Symptomatic and Idiopathic Fever.*

1st. If the fever be symptomatic of inflammation, the quantity of fibrin is augmented; but this increase does not depend on the fever, for there are several fevers, just as intense and long continued as the inflammatory, in which we observe no increase of fibrin; the cause of the increased quantity of fibrin is not easily discovered, and to assign any particular one, in the present state of our knowledge, would be hasty.

2nd. When the fever does not depend on inflammation, we have no augmentation of fibrin, be it ever so severe or prolonged.

3rd. Should simple fever become complicated, during its course, with any inflammatory affection, then the fibrin increases.

4th. Fever and inflammation may co-exist, the latter being an essential element of the former, as inflammation of the skin in small-pox, measles, scarlatina, &c.; these affections of the skin, which perhaps should not be ranged under inflammations, are unattended with the characteristic increase of fibrin; the same remark applies to ulceration of the intestines in typhoid fever; however tenaciously some physicians may adhere to the inflammatory doctrine of typhoid fever, it is certain that the ulcerations of the intestinal glands are not accompanied by that increase of fibrin which attends other inflammatory diseases. Hence, whenever fever co-exists with inflammation, and together with it constitutes one of

* Prov. Med. and Surg. Journal, Aug. 1841.

the elements of the disease, the fibrin of the blood is not increased; for example, small-pox, typhoid fever, &c. But it is far different when inflammation springs up during the course of the fever, or is one of its effects.

In a certain class of fevers, the chief cause seems to be the excessive richness of the blood. Inflammatory fevers, of a few days' duration, depend upon this cause.

This richness consists in an increase of the globules, not of the fibrin. The same increase occurs at the commencement of typhoid fever, measles, and scarlatina. We do not find this augmentation of globules in inflammation; it exists in the middle period of fevers.

In continued fevers, the febrile movement persists even when the globules have fallen to their normal standard, or below it. M. Andral instances chlorosis.

Fever may exist with a normal state, increase, or diminution of the globules. We find that the simple presence of fever never determines an increase in the quantity of fibrin; that fever may exist; 1st, when both fibrin and globules are in normal quantity; 2nd, when the globules alone are increased, the fibrin remaining unchanged. The quantity of fibrin may fall, during typhoid fever, even so low as 0.9; at an early stage of the disease, or when it is mild, the fibrin remains unchanged, but falls as the fever is aggravated; when it assumes an ataxic character, and symptoms of prostration ensue about the fifteenth day, the fibrin also falls; but this is not the case with inflammation.

2. *State of the Blood in Inflammation.*

The condition of the blood in inflammation differs according as the latter is acute, subacute, or chronic.

*Fibrin.*—The fibrin is always increased during inflammation; it may vary from 4 to 10.

*Globules.*—These are not necessarily augmented; generally speaking, they retain their normal standard; in rare cases are increased; in others diminished. As the inflammation advances the globules may fall, but this is the effect of blood-letting and abstinence. We have already seen that plethoric persons are not more disposed than others to inflammation.

*Solid contents of serum.*—The albumen may be increased, but not necessarily, and the inflammation may attain a very high degree of intensity, without augmentation of the serum.

*Physical properties.*—The clot is generally very firm and tenacious, because the fibrin has expelled a great portion of the serum; in fever, on the contrary, the serum is retained, and renders the clot soft and voluminous. In inflammation the clot is small: for as it contains a large proportion of fibrin, the globules are firmly pressed together by the contraction of the coagulating part of the blood.

3. *Nature of False Membranes and Pus.*

Whenever inflammation terminates in suppuration, the quantity of fibrin increases; hence, the formation of pus and augmentation of fibrin accompany one another; we might, indeed, add another phenomenon, viz. the formation of false membranes. The fibrin is also augmented in cases where the serum is turbid and mixed with flocci; on analysing false membranes they are found to be composed of fibrin, and this is confirmed by a comparison of false membranes with the buffy coat of the blood, to which they bear a perfect resemblance.

Pus is a compound fluid, the composition of which is not yet accurately known; we are unable to assert that it is formed of fibrin, but in certain kinds of pus we find a white substance analogous to fibrin.

---

### BETHLEM HOSPITAL.

### Report of Sir Alexander Morison—Pathology of Insanity.

This Report furnishes an account of the post-mortem appearances presented by the patients who died in the hospital during the last five years. The number of deaths amongst the females was nineteen; amongst the males, twelve.

*Morbid Appearances in Females.*

*Case* 1.—No deviation from the normal condition of the brain and membranes observed except congestion of the blood-vessels, both external and internal; the cerebral substance, the ventricles, and the arachnoid, were perfectly healthy. The left lung was hepatized, and marks of disease were observed in the chest and abdomen.

*Case* 7.—The blood-vessels of the brain and membranes turgid; the cellular texture of the pia mater on the convexities of the cerebral hemispheres largely infiltrated; the fluid in the lateral ventricles increased in quantity; there was much fluid in the cranium after the brain had been removed.

*Case* 8.—The convolutions of the cerebral hemispheres were partially flattened; the blood-vessels of the brain and membranes were loaded; when the dura mater was divided and detached, the subjacent membranes exhibited three or four small patches of a bright yellow discoloration, but no fluid could be squeezed out of them; the cut surfaces of the cerebral substance everywhere exhibited numerous bloody points; the lateral ventricles were distended with about two ounces of turbid fluid in each; there was thick yellow pus, about one or two tea-spoonfuls, in the bottom of the reflected horns of each ventricle; the lining membrane of the ventricles exhibited vascular ramifications and minute ecchymoses, and the arachnoid coat covering the pons varolii and neighbouring parts of the brain was thickened and opake, and of a light yellow colour from purulent infiltration; the substance of the brain was soft, particularly around the ventricles and at the basis. The cause of these appearances is conceived by Mr. Lawrence, to whom I am indebted for the description of the morbid appearances, to have been acute inflammation of the lining membrane of the ventricles and of the arachnoid coat.

*Case* 15.—Much blood escaped on dividing the integuments and sawing the skull, and the vessels of the brain and membranes were enlarged. Five or six quarts of fluid of a reddish colour were contained in the chest.

*Case* 17.—The blood-vessels of the brain and membranes were turgid; in other respects the contents of the cranium appeared healthy; marks of inflammation were visible in the pleura, in the cavity of which bloody fluid was contained.

*Case* 18.—A large quantity of blood escaped from the vessels of the head in cutting the skin and sawing through the skull; the vessels of the brain were moderately injected, and there was slight serous infiltration of the pia mater; in other respects the contents of the cranium were perfectly healthy, as also those of the thorax and abdomen.

*Case* 19.—In this case there was general fulness of the blood-vessels; sections of the cerebral substance everywhere exhibiting numerous divided orifices; there was serous infiltration of the pia mater; at some points of the cerebral hemispheres the convolutions were shrunk so as to leave conspicuous intervals, which were occupied by the infiltrated pia mater; the substance of the brain appeared to be healthy and firm; the trachea and larynx, the contents of the chest, and of the abdomen, were all perfectly healthy, exhibiting no appearance to throw any light on the very sudden death of this patient, which it was imagined might have proceeded from an affection of the heart or some large blood-vessel.

*Morbid Appearances in Males.*

*Case* 3.—Remarkable turgidity of the blood-vessels, in the substance of the brain especially; the superior longitudinal sinus filled with a coagulum firmly adhering to its sides like a recent clot, at two or three points gradually changed into a dull reddish brown fluid, of the consistence of pus; a large vein about the middle of each hemisphere greatly distended, and filled with a fine coagulum, presenting at some points a similar fluid; this vein terminated at each side in the cavernous sinus; other veins were filled with firm coagula; a considerable ecchymosis of the pia mater, and slight infiltration of that coat. The lungs were in parts hepatized, and contained an abscess.

*Case* 5.—Blood-vessels of the brain and membranes turgid; numerous bloody points in the cerebral substance, and the medullary matter presenting here and there a faint violet tint; slight serous infiltration of the pia mater in the cerebral hemispheres; about an ounce of clear fluid in each lateral ventricle. The lungs were diseased.

*Case* 6.—The blood-vessels of the brain and membranes extremely turgid; the cellular texture of the pia mater in a state of serous infiltration over the entire upper and lateral surfaces of the cerebral hemispheres; the lateral ventricles contained rather more than the normal quantity of fluid, and there was much fluid in the basis of the skull. Extensive hepatization, with a large abscess in the lungs.

*Case* 7.—The arachnoid coat somewhat thickened and opake, and the pia mater considerably infiltrated over the cerebral hemispheres: the lateral ventricles enlarged, and filled with transparent fluid; a considerable quantity of fluid in the basis of the skull; no deviation from the healthy state observed in the substance of the brain.

*Case* 8.—The blood-vessels of the brain and membranes turgid; numerous bloody points appeared in every situation; the arachnoid coat thickened and partially opake, especially along the edges of the fissure between the cerebral hemispheres; the cellular substance of the pia mater in the hemispheres considerably infiltrated. The structure of the brain appéared natural.

The mucous membrane of the trachea and bronchii of a bright red, and covered with a thick yellow secretion; the lungs adhered to the sides in several places; contained an abscess and an enlarged bronchial gland containing a substance like putty.

*Case* 10.—The blood-vessels of the brain and membranes were turgid; the arachnoid coat on the cerebral hemispheres was considerably thickened and opake; there was great infiltration of the pia mater, and an increased quantity of fluid in the ventricles.

---

### Clinical Remarks of M. Velpeau.*

### 1. *Cause of Metastatic Abscesses.*

On one point, however, authors are not well agreed; and that is the manner in which the pus becomes mixed with the blood. Marechal, Legallois, Rochoux, and others, think that it may be explained by venous absorption from the surface of wounds; others, as Dance, Blandin, Arnott, &c., assert that the presence of pus is always preceded by inflammation of the veins, which produces the matter found in the circulation or tissue of organs after death; according to the latter authorities, pus cannot be transported from one part of the body to another without undergoing decomposition. Hence, gentlemen, we have two theories of

* Provincial Medical Journal, Aug. 14, 1841.

metastatic abscess: one consisting in simple absorption from the surface of wounds; the other in inflammation of the veins. For my part, I feel convinced that inflammation of the vein does not constantly exist, but that the affection may be produced either as a consequence of phlebitis or of simple absorption. I perfectly agree with Dance, Berard, and Blandin, as to the pernicious effects of phlebitis on the blood; but I differ from them completely in this, that I do not admit phlebitis to be the primitive or even frequent cause of metastatic abscess; the veins, it is true, are frequently inflamed, and may, in certain cases, be the cause of purulent absorption; but in many others we have no inflammation of the veins, and the pus may be introduced into the torrent of the circulation either by the lymphatics, the veins which open on the wound, or by imbibition. How often have I found large collections of pus in the viscera, without being able to detect the slightest trace of inflammation in any part of the venous system? Upon this point I am positive, having determined it so frequently by the most careful examination.

2. *Metastatic Abscess not always fatal.*

We must not, however, completely despair of saving the patient; for when the unfavourable symptoms last for two or three days only, or when they terminate in some crisis by the urine, general perspiration, &c., and the febrile symptoms subside, some hope is left; I have seen several patients recover under these circumstances; however, we must allow that such cases are very rare.

3. *M. Velpeau's Treatment of Metastatic Abscess.*

Endeavour, in the first place, to determine the fluids towards the wound by large poultices; at the same time apply blisters on the legs or thighs; give some diuretic tisan internally; if the patient be young and robust, the pulse full and strong, then bleed; if he complain of severe pain in the chest or abdomen, apply some leeches or cupping-glasses. Should the wound present a dry, unhealthy aspect, you may employ a bark lotion, or in certain cases apply leeches, or scarify it, or put on a blister; these means are particularly indicated when you have any reason to suspect the existence of phlebitis. You may also envelop the limb in a bandage from the wound towards the trunk, so as to exercise powerful compression; purgatives, also, should be employed in addition to the means now pointed out. Should stupor, with meteorismus and dark incrustation of the mouth exist, you may try tartar emetic in high doses. When the patient is very feeble, give the bark or the sulphate of quinine, especially in cases where the symptoms are intermittent. Of the local means, there is none in which I have so much confidence as the bandage, if applied before the pus has found its way in any quantity into the circulation. In inflammation of the veins of the extremities, when the disease has not ascended beyond the venous radicles, and before the formation of metastatic abscess, we can almost always arrest it by the bandage; even when the blood has been tainted, we may still employ it, because we thus cut off the source of the poison, and give the vital powers a chance of overcoming the malady.

We confess that we feel doubtful, at all events in many cases, of the propriety of compression. In a large proportion of instances the primary injury scarcely admits of *such* compression *as can cut off* the entrance of the pus into the circulation; and if it is not cut off, we are unable to see what is gained. Metastatic abscess follows compound fractures of the lower limbs as frequently as any other local lesion. How is compression to prevent the entrance of pus into the veins and absorbents? How indeed is it to do so in any case, for compression which should go to that extent must do infinite mischief to the part.

The remedy from which we have seen most service has been blistering. A very bad case did well under the application of repeated blisters, and in another case the abscesses disappeared with a large deposition of purulent matter in the urine.

### CONCLUSIONS OF M. CRUVEILHIER WITH REGARD TO THE SOUNDS OF THE HEART.

It seems, from some experiments which M. Cruveilhier made, that the two sounds of the heart arise at the roots of the aorta and pulmonary artery, and depend on the motions of the sigmoid valves; that the first sound which corresponds to contraction of the ventricles depends on the repression of the sigmoid valves; that the second sound, accompanying the dilatation of the ventricles, is caused by the spreading out or expansion of the same valves by the retrograde column of blood.

---

### NATIVE GENERAL HOSPITAL OF BOMBAY.

### ANNUAL REPORT FOR THE YEAR 1839.*

1. *Narcotine in Intermittent Fevers.*

A portion of narcotine, procured from Bengal, had a fair trial in thirteen cases, viz. five tertian and eight of the quotidian type, and only succeeded in three—one tertian and two quotidian. The remedy was given in an acid solution in ten grain doses at intervals of two hours for four times; in all of these cases, it even, where it succeeded, produced giddiness, nausea often followed by vomiting and great gastric uneasiness; and difficulty was often experienced in inducing patients to swallow this remedy, who took quinine most readily and without complaint. I think, I noticed that, even in the three successful cases, anorexia was present to a greater extent, and continued for a longer period than seemed likely to have been the case, had narcotine not been used for their cure; ten grain doses were had recourse to, from smaller having been found quite inefficacious. In three cases I gave the medicine in fifteen grain doses, but in all, the first dose was followed by great uneasiness at stomach, and the second was invariably followed by vomiting. Quinine cured all those cases, but in two of them, not till after a long period, and only in large doses (ten grains in each). I may remark that the narcotine was tried by Dr. O'Shaughnessy's test, and found by it, to be perfect.

2. *Small-pox after Vaccination.*

Cases of this disease occurring in parties unprotected by vaccination of unusually severe form, have been admitted in every month of the past year, excepting in January, February, and October. The greatest number of admissions and the smallest mortality occurred in March, when many recently attacked were admitted into hospital; but in the other months the cases received have been of the worst possible description, and always at advanced periods of the disease. A confluent rash, and great affection of the throat and head in most, and a copious but imperfectly developed rash in others, have been the most marked features of all the cases.

3. *Recovery from penetrating Wound of Thorax and Abdomen.*

The injuries were inflicted by a man upon his wife. Both blood and air escaped from the wounds of the chest, and a portion of the omentum (which was removed) had escaped from the abdomen; in addition she had a long deep wound at the angle of the jaw, her mouth laid open by another of the cheek, and eight other deep flesh wounds of the trunk and extremities; and yet has never had, after the first thirty-six hours, an unfavourable symptom, and has merely required the exhibition of anodynes, antimonials, and laxatives.

---

* Transactions of the Medical and Physical Society of Bombay.

4. *Traumatic Tetanus cured.*

The three cures were cases of traumatic tetanus, and in all salivation was produced, after which the symptoms gradually yielded. A case of severe opisthotonos from Guinea-worm, which had appeared at three different parts in the soles of the feet and legs, is now under cure. The patient was admitted on the 4th December, fifteen days after the first appearance of the tetanic symptoms, and in a state which seemed to preclude the possibility of his living many hours. A strong cathartic was given on admission, followed immediately afterwards by a five minim dose of hydrocyanic acid combined with tinct. opii ʒij. This seemed to give relief, and was soon repeated in an increased dose, and he at length took ten minims of the acid with ʒij. of tinct. opii four times a day with occasional purgatives, with the most marked benefit. He can now, 1st January, walk a little, has no rigidity of limbs, and only feels very weak. The acid has been intermitted, and he now only takes half a drachm of tinct. opii twice daily.

---

### Annual Report of the Diseases in the Jail and Detachment Hospitals at Rutnagherry.

1. *Curious Course of a Bullet.*

A man was shot in the back near the angle of the ribs, the ball taking an oblique direction towards the spine: it could not be extracted, and he died, reduced to a skeleton, after being three months in hospital. From the curvature of the spine which took place to a great extent before death, it became evident that there was a loss of substance in the body of one or more of the vertebræ, and accordingly, on dissection, the ball was found to have passed through this part of the bony column, and was discovered lying behind the right kidney, much flattened and indented. The bodies of the two lower dorsal, and of the two upper lumbar vertebræ were almost entirely removed by the passage of the ball, and by subsequent caries and absorption. The escape of the aorta, and the spinal cord and its coverings in such an injury, was a singular feature of this case.

2. *Scurvy.*

The prisoners at Rutnagherry were subjected to unusully rigorous confinement, and the consequence was scurvy. Dr. Bourchier gives a good account of the symptoms.

The disease generally made its attack by a swollen and spongy state of the gums, which were more or less of a livid colour, and bled readily on being irritated; in many instances they extended in triangular processes between the teeth, or projected over them; the tongue sometimes, but not invariably, had an excoriated or fissured appearance near the tip; in seven cases, the mucous membrane of the cheek presented a similar appearance. The breath was generally fetid, and the face more or less puffed and swollen. The affection of the mouth was attended by various degrees of pain and swelling of the lower extremities, most frequently of the hams, in which hard tumors formed.

When the scorbutic diathesis was established, the mere pressure of the irons was apt to produce swelling and inflammation. In one or two cases, very acute pain was felt in the muscular parietes of the chest, unattended with swelling. Lind calls this "a dangerous symptom." It was observable that the greatest swelling was by no means attended with the greatest pain, the legs and ankles being often affected with an enlargement of a stony hardness, scarcely at all painful, and chiefly inconvenient by preventing the play of the muscles and action of the joints; whereas in a few cases the patients suffered most acutely from the slightest pressure on the lower extremities when scarcely at all swollen. In such, the parts had more of a shining appearance, and were much less rigid. The affection of the mouth usually appeared first, but sometimes the scorbutic swellings of the limbs preceded it. The constitutional disturbance was not very considerable in the generality of cases, but lassitude and depression of spirits in various degrees, accompanied the local symptoms, and the countenance exhibited a degree of dejection more or less marked.

The most successful treatment consisted in the exhibition of tonics and cordials, including the bark in decoction and in powder, and particularly quinine. Vegetable acids, as limes and tamarinds, were very freely employed, in combination with "*goor*," or coarse sugar, in itself an excellent antiscorbutic. The local applications which were of most service, were washes and gargles of alum and decoction of bark, and the solution of chloride of lime, hot fomentations and friction (when the case admitted of it) with camphor liniment, or turpentine and oil, to the swellings on the limbs, and opiate plasters when the severe pain would admit of nothing else. With these measures, a more liberal diet than usual was allowed, and the general result was highly satisfactory.

Of the sixty-nine cases, three terminated fatally, two of them being old and debilitated subjects—the third was a robust man, who seemed improving, when he was suddenly attacked with extreme oppression at the chest, and died in a few hours in spite of every thing that could be done. Æther, camphor and opium, with a blister to the chest, gave temporary relief, but he died, after great suffering, with apparent symptoms of effusion on the chest.

Dr. Bourchier has tried, but without satisfactory results, the nitrate of potash. It failed in his hands.

3. *Inveterate Itch cured by Bichloride of Mercury.*

One inveterate case of vesicular itch, incurable by the ordinary means, yielded to a solution of the bichloride of mercury, in the proportion of ʒi. to a pint of water, without any unpleasant effects being occasioned by the remedy.

---

### GUY'S HOSPITAL.

### TREATMENT OF FISTULA LACHRYMALIS.*

In cases in which fistula lachrymalis is threatened, the great thing is to keep a free passage through the nasal duct. Mr. Morgan recommends the plan he has been in the habit of pursuing.

As a preventive measure, the mechanical removal of all impediment in the duct is as urgently called for, when the sac is simply distended by its contents in the onset, and before the part is acutely inflamed, as it is when an abscess has formed in that part, the bursting or opening of which affords the surgeon an opportunity of passing his probe with ease through its interior into the strictured canal. Now, gentlemen, I treat a case of stricture in the nasal duct upon the same principles, and nearly in the same manner, as I do a case of stricture in the urethra. In both cases, we find a contraction in the calibre of an excretory duct, resulting from the same cause, viz. an interstitial effusion of adhesive matter, the consequence of inflammation; and in both cases, the object we have in view is to dilate the stricture without lacerating, or in any way injuring, adjacent tissues. In cases of stricture in the urethra, we do not wait for an opportunity of passing an instrument through the contracted canal, till an abscess in the perineum brings us into immediate contact with the disease; nor do we cut down upon the membranous portion till other means have failed; and why should we wait for an abscess in the sac, or open that part through the integu-

* Prov. Med. Journ. Aug. 28, 1841.

ments of the face, with a view of dilating the nasal duct through its interior, when the same mode of treatment as that which we pursue for the cure of stricture in the urethra, is equally available for the cure of stricture in the ductus ad nasum? Through the orifice of this duct, where it enters the nose, a probe can by a practised hand be almost as readily carried through its canal to the stricture, as the bougie is passed through the orifice of the urethra to its membranous portion; and it is by availing ourselves of this mode of applying the mechanical means of remedy, that we shall be enabled to remove at once the greatest difficulty we have to contend with in the treatment of the diseases which I am now describing. The only objection which can be offered to the plan I now recommend to you, must, I should think, be the difficulty of passing the instrument; but this objection ought never to be raised, for experience will prove to any one who will take the trouble of trying the experiment, that a little practice will enable him to acquire sufficient dexterity to perform this operation with ease. This may be done by rehearsing it, whenever opportunity offers, upon the dead subject, upon which, as I have now one before me, I will point out the proper mode of proceeding. The instrument I use, and which I now show you, is a metallic bougie, or sound, rather thicker than a common probe, and somewhat conical at the point, bent, as you see, in the shape of an italic letter *S*, for I find this the most convenient form; the handle is flattened, and the instrument about three inches in length. I first pass the point along the floor of the nose, keeping it downwards at first, afterwards carrying it outwards, till I find I have placed it between the inferior turbinated and superior maxillary bones. I then turn it directly upwards, and in this way introduce it into the termination of the duct, which can generally be distinctly felt, as the point is moved gently backwards and forwards along the membrane. This is the most difficult part of the operation, for when you have once fairly entered the point, it is easily pushed onwards by depressing the handle of the instrument. Great care must, however, be taken not to use force in passing it through the duct, the bony case of which is very easily broken down; if you meet with much resistance, which you will seldom do, unless in chronic cases, you must satisfy yourself at first with merely entering the point into the stricture, from time to time, until the constricted parts have yielded; and while you are thus endeavouring gradually to dilate the stricture, as well, indeed, as during the whole process of cure, even when the instrument has been passed, you will find very great advantage from the injection of tepid water in the acute, and astringents in the chronic stages, through the canal; this is best effected by introducing the instrument I now show you into its nasal termination. You see that it is a small catheter of the same shape as the metallic bougie, through which, by attaching a syringe to one end, an injection can be thrown with sufficient force to wash out thoroughly both the duct and sac; a part of your treatment which is of considerable importance. In adopting this mode of proceeding, it is unnecessary to push the end of the catheter into the sac, for if it is merely introduced into the mouth of the duct, the injected fluid will readily pass into the inflamed cavities. You must, I think, be at once convinced of the superiority of this plan, when it is practicable, over that which is usually adopted,—I mean the injection of the lachrymal conduits through the puncta. Sometimes, however, when the stricture is firm, and the canal perfectly impervious to the passage of either your instrument or your injections, you must have recourse to this latter mode for accomplishing your object, and, by introducing a punctum tube, inject the sac by means of Anel's syringe. You are always to fill and empty this two or three times by pressure, whenever you use the injection. If you are called to a case of abscess of the sac presenting an external opening, it is better to pass your probe first through the upper opening of the duct; this is of course by far the most easy way of overcoming the obstruction; and having once passed a probe in this direction, its introduction from below is at once insured, and you may venture to use more force in operating through the sac than through the nose.

Having taken advantage of an opening thus already made, by establishing the continuity of the canal in this way, it will be your object to prevent a fistulous opening in the face, from the continued discharge of the abscess, by endeavouring to effect the closure of the wound, avoiding the subsequent use of any instrument through the sac, and insuring the permanent dilatation of the stricture, by carrying on your operations from the nose. The nasal probe or bougie should be used about twice a week, and the sac and duct injected daily. In those cases where abscess has formed, and the integuments have become not only deeply discoloured, but obviously so thinned by absorption, as to render the natural evacuation of pus through the surface inevitable, it is better to puncture the abscess at once, and having passed a probe through the duct from the sac, endeavour to close the opening in the face by following the plan of treatment I have mentioned, and which, perhaps, if pursued in the commencement, might have prevented the disease from terminating its course in the manner I have described. Keep the nasal duct freely open, gentlemen, and you need not fear fistula lachrymalis, or abscess of the lachrymal sac.

---

### BRUIT DE SOUFFLE IN ANÆMIC CASES.

The following observations, and they are valuable, will be found in M. Andral's Lectures.

1st. When a patient has been bled several times, or suffered from frequent hæmorrhage, the *bruit de souffle,* either permanent or intermittent, often occurs, but not constantly.

2nd. In spontaneous anæmia the same bruit exists.

3rd. Whenever the blood globules fall below 80, the *bruit de souffle* is constantly heard in the arteries, and sometimes in the heart.

4th. The same often occurs with diminution of the globules between 80 and 100; or in rarer cases, between 100 and 125.

5th. In some very rare cases the *bruit de souffle* exists when the globules are at 131 to 137; hence we must conclude that it is not exclusively connected with diminution of the globules.

The above results were obtained from an analysis of 93 cases, in which the *bruit de souffle* existed in the heart or arteries. Amongst the 93 cases it was permanent in 56; and in the latter the globules were 28 times above 80; 13 between 80 and 100; 10 between 100 and 115; 5 between 115 and 125. It was intermittent in 3 cases, where the globules were below 80; in 13 between 80 and 100; in 8 between 100 and 115; in 5 between 115 and 125; in 3 between 125 and 137; and in 3 between 131 and 137. The intermittent souffle is less valuable as a symptom than the constant; the latter is more characteristic of diminution of the globules, and becomes intermittent as their proportion is increased.

The *bruit de souffle* occurs in several other diseases, as well as in chlorosis. In one case of rheumatism, where the globules were at 97, the permanent bruit was heard; it was intermittent in another case, where they were at 99; from 81 to 97, it was sometimes permanent, sometimes intermittent. This bruit very rarely occurs in pneumonia, although the patient may have been frequently bled; a circumstance readily explained by the inflammatory nature of the disease; but it should be remarked, that the globules never fall as low in pneumonia as in rheumatism. From the above, it is evident that abstraction of blood is injurious in persons of anæmic temperament, unless there exist some disease which imperatively demands it; on the contrary, substantial diet augments the globules, and hence is beneficial; the same remark applies to the preparations of iron, which increase the proportion of globules, and not the fibrin.—*Prov. Med. and Surg. Journ. Aug. 7,* 1841.

